# Nitrogen Isotope Fractionation During Nitrate and Ammonium Uptake in Maize: Hydroponic Evidence and Implications for Ecological Investigations

**DOI:** 10.1111/pce.70330

**Published:** 2025-12-19

**Authors:** Priscillia Semaoune, Joëlle Templier, Sylvie Derenne, R. Dave Evans, Mathieu Sebilo

**Affiliations:** ^1^ Sorbonne Université Paris Cedex France; ^2^ School of Biological Sciences and WSU Stable Isotope Core Laboratory Washington State University Pullman WA USA

**Keywords:** isotopic fractionation, maize, NH4+ uptake, NO_3_^−^ uptake

## Abstract

Understanding nitrogen (N) isotopic fractionation during plant uptake is critical for interpreting δ^15^N variations in terrestrial ecosystems. We investigated isotopic discrimination during ammonium (NH_4_^+^) or nitrate (NO_3_^−^) uptake in maize (Zea mays) grown hydroponically under controlled conditions with 0.2 and 2 mM to represent high and low affinity transport systems, respectively. Nitrogen (^15^ε) and oxygen (^18^ε) isotopic fractionation during NO_3_^−^ uptake were determined. NO_3_^−^ uptake exhibited low and concentration‐independent ^15^ε values (0.2 mM: ^15^ε = −2‰; 2 mM: ^15^ε = −1.7‰). In contrast, ^18^ε was lower at high concentrations (0.2 mM: ^18^ε = −5.3‰; 2 mM: ^18^ε = −2.1‰). For NH_4_^+^ uptake, ^15^ε was higher and increased with concentration (0.2 mM: ^15^ε = −5.7‰; 2 mM: ^15^ε = −8.5‰). An isotope mixing model suggests a small NO_3_^−^ efflux contributes to ^15^N and ^18^O enrichment in solution due to significant isotopic fractionation during assimilation. The discrimination between source and plant δ^15^N is influenced by the source δ^15^N, the magnitude of ^15^ε, N supply, and uptake kinetics. While plant δ^15^N integrates source δ^15^N over time, it is unsuitable as a direct tracer. This study refines the understanding of isotopic fractionation mechanisms in plant N uptake and their implications for δ^15^N‐based ecological investigations.

## Summary statement

Controlled hydroponic experiments reveal distinct nitrogen isotope fractionations during nitrate and ammonium uptake in maize, refining our understanding of δ¹⁵N patterns in ecological contexts.

## 1 Introduction

Nitrogen (N) is an essential element for plant growth, crop productivity, and ecosystem functioning (Anas et al. 2020). Its availability in soils controls key ecological processes and regulates primary productivity in both natural and managed systems (Lü et al. 2014). Plants primarily take up inorganic forms of N‐ ammonium (NH_4_^+^) and nitrate (NO_3_^−^) that originate from organic matter mineralization, fertilization, and atmospheric deposition (Galloway et al. 2003; Kalcsits and Guy 2016). These two N forms differ in solubility, mobility, uptake pathways, and assimilation biochemistry, leading to contrasting physiological responses and nitrogen‐use efficiencies (Xu et al. 2012). In soil solution, the concentration of plant‐available inorganic N can range from micromolar to millimolar (Glass et al. 2001). Consequently, both the form and concentration of N may influence plant isotopic composition.

Nitrogen isotope composition (δ^15^N) provides a valuable integrative tool to investigate N sources and transformations within plant‐soil systems. Isotopic fractionation accompanies nearly all steps of nitrogen cycle‐mineralization, nitrification, denitrification, uptake and assimilation‐causing marked variability in the δ^15^N of plant‐available N (Evans 2007; Craine et al. 2018). Accordingly, δ^15^N measurements in plants have been widely used to trace N cycling and assess N source utilization across ecosystems (Michelsen et al. 1996; Miller and Bowman 2002; Falkengren‐Grerup et al. 2004; Bai et al. 2008; Kahmen et al. 2008). However, isotopic discrimination during N uptake and internal assimilation can significantly decouple δ^15^N from that of the source, complicating interpretation if these processes are not well constrained (Evans 2001; Kalcsits et al. 2014). Substantial isotopic fractionation during N uptake could confound interpretation of δ^15^N variations in plants and their N sources if not properly accounted for. Understanding when and to what extent isotopic fractionation occurs during N uptake is crucial to accurately link the isotopic composition of plants to their N sources (Kalcits et al. 2014).

Few studies have investigated isotopic fractionation associated with N uptake under controlled conditions on a single N source, and these experiments have yielded contradictory results. When NO_3_^−^ was used as the sole N source across different plant species, some studies reported no isotopic N fractionation over a wide range of concentrations (Mariotti et al. 1982; Yoneyama and Kaneko 1989; Evans et al. 1996), while others observed fractionation that depended on concentration (Mariotti et al. 1982; Högberg et al. 1999; Kolb and Evans 2003) or was independent of it (Yoneyama et al. 2001). Similarly, NH_4_^+^ uptake as a sole N source has been associated with either no or minimal isotopic fractionation (Evans et al. 1996; Kolb and Evans 2003), or with a larger isotopic fractionation that increases with NH_4_^+^ concentration (Yoneyama et al. 1991, 2001). Three potential mechanisms have been suggested to explain the observed variation in isotopic fractionation during N uptake. (1) The involvement of distinct N forms‐ and concentration‐dependent carrier systems, which may discriminate differently against ^15^N and induce different isotopic fractionation patterns. The mechanisms of N uptake in plants vary depending on the concentration of N available in soil. Physiological studies indicated that both NO_3_^−^ and NH_4_^+^ uptake involve high and low affinity transport systems, with transitions occurring at concentration below and above 0.5 mM (Aslam et al. 1992; Glass and Siddiqi 1995; Crawford and Glass 1998). (2) The efflux of ^15^N‐enriched inorganic N from roots into the medium which can alter the isotopic composition of the remaining N pool. Enzymes involved in NO_3_^−^ and NH_4_^+^ assimilation discriminate against ^15^N (^15^ε = −15‰) (Ledgard et al. 1985; Yoneyama et al. 1993), leading to ^15^N enrichment in the unassimilated inorganic pool within roots, while organic N products become depleted in ^15^N. High influx of inorganic N into plants, exceeding enzymatic demands, has been proposed to cause the efflux of ^15^N‐enriched inorganic N from roots (Mariotti et al. 1982). This hypothesis has been tested by comparing wild‐type plants with nitrate reductase (NR)‐deficient mutants (Yoneyama et al. 2001; Kolb and Evans 2003). In the absence of NR, assimilation and therefore isotopic fractionation would not occur, and any efflux of unassimilated NO_3_^−^ from roots would retain the same composition as the source solution. No differences in isotopic fractionation during uptake were observed, and the δ^15^N of unassimilated NO_3_^−^ in roots for mutants was identical to that in wild‐type plants (Evans et al. 1996; Yoneyama et al. 2001). It has been suggested that assimilation occurs in NR‐deficient mutants due to leaky mutations or the presence of an unidentified NO_3_^−^ reducing enzyme (Kolb and Evans 2003). (3) For NH_4_^+^, the volatilization of ^15^N depleted‐NH_3_ under high NH_4_^+^ concentration and elevated solution pH can significantly enrich the remaining NH_4_^+^ in ^15^N (Ariz et al. 2011). Strictly defined, ‘uptake’ refers to the influx of N via transporters within the plant. However, when processes such as efflux (2) and volatilization (3) are considered, net uptake is defined as the combined result of these processes (Ter Steege et al. 1999).

To enhance understanding of the physiological processes underlying the discrimination between plant and source δ^15^N, this study investigated N isotopic fractionation associated with NO_3_^−^ or NH_4_^+^ net uptake in hydroponically grown maize. A closed system nutrient supply was used, characterized by the absence of substrate renewal, allowing reactions to occur sequentially over the time, as the substrate was gradually consumed. As fractionation occurs, the δ^15^N of the substrate increases while the N concentration decreases. The study tracked the isotopic compositions of both N and O in the source and plants throughout NO_3_^−^ uptake kinetics.

In this study, we quantify nitrogen isotope fractionation associated with NO_3_^−^ and NH_4_^+^ uptake by axenic maize (*Zea mays* L.) grown hydroponically at two external concentrations (0.2 and 2 mM). We combine δ^15^N measurements of plant tissues and nutrient solutions with δ^18^O analyses of NO_3_^‐^ and isotope mass‐balance modeling to (1) determine ^15^ε for ammonium and nitrate uptake under contrasting N supply regimes, (2) evaluate the roles of assimilation localization and efflux in generating intra‐plant δ^15^N differences, and (3) assess how initial plant N stocks and uptake kinetics influence the relationship between source and tissue δ^15^N. By identifying these processes under controlled conditions, we aim to clarify the physiological basis of isotopic discrimination during N uptake and to provide a mechanistic reference framework for interpreting plant δ^15^N patterns in the environment.

## 2 Materials and Methods

### 2.1 Pretreatment

Seeds of *Zea mays* L. were sterilized by immersing them successively in 96% C_6_H_6_O for 5 min and 30% H_2_O_2_ for 5 min, followed by thorough rinsing with deionized water. Seeds were germinated in tubs filled with glass beads that were moistened with deionized water at 23°C in dark. Tubs and glass beads were sterilized under ultraviolet light for 5 h prior to use microbial growth. Seven days after germination, the seedlings were supplied with a modified Hoagland solution containing either 0.2 mM NH_4_^+^ or NO_3_^−^. After 5 additional days, the seedlings were transferred to hydroponic systems housed in a Plexiglas chamber with controlled light and temperature conditions. The chamber was maintained at a constant temperature of 25°C, with a 12 h photoperiod and a light intensity of 400 µmol photons m^−2^ s^−1^. Three 5‐liter containers were used for each treatment concentration (0.2 and 2 mM) and placed in the chamber. Separate experiments were conducted with NH_4_^+^ and NO_3_^−^. Each container housed 5 plants. Foam plugs were wrapped around each plant at the root‐shoot junction and fixed into holes in the lid of the containers. A consistent ratio of 1 L of solution per plant was maintained throughout the experiment. During the growth period, the nutrient solution was maintained at the treatment concentrations of 0.2 or 2 mM of NH_4_^+^or NO_3_^−^. The nutrient solution was changed one or two times per week for the 0.2 and 2 mM treatments, respectively. The pH of the nutrient solution was monitored daily and maintained between 4 and 5 throughout the experiments. The solution was bubbled with air 1 h per day to ensure an adapted oxygenation and homogenization of the nutrient solution. Denitrification can be excluded because the medium was aerated and no nitrites (NO_2_^−^) were detected in nutrient solutions. Similarly, nitrification didn't occur, as no NO_3_^−^ was observed in NH_4_^+^ nutrient solutions.

### 2.2 Experimental Protocol

At 28 days old, when the plants had developed four leaves, the roots and the containers were rinsed with deionized water and fresh nutrient solutions containing 0.2 or 2 mM of NH_4_^+^ or NO_3_^−^ were added. At the beginning of the experiment, one plant and one liter of nutrient solution were collected from each container and this procedure was repeated at subsequent time points during the uptake kinetics. Preliminary tests were conducted with the same species to estimate uptake rates and ensure appropriate sampling throughout the kinetics. In total, 5 measurements were performed during the uptake kinetics. Maintaining a ratio of 1 L per plant was crucial to preserve the integrity of the closed system. During harvesting, the roots were quickly rinsed with deionized water and the plants were separated into roots and shoots. The plant materials were dried in an oven at 50°C for 2 weeks and their dry biomass was determined. The dried tissue samples were ground into a fine powder. NO_3_^−^ was extracted from the tissue samples using hot water, following the method described by Evans et al. (1996). Nutrient solution samples were immediately frozen until analysis. At each time point during the kinetics, the δ^15^N and δ^18^O values of NO_3_^−^, δ^15^N of NH_4_^+^ in nutrient solution, and the δ^15^N of total N in plant tissues were measured. The δ^15^N and δ^18^O values of NO_3_^−^ extracted from the plant tissues were measured only for the first and the final time points of the uptake kinetics.

### 2.3 Analysis of N Content and Stable Isotope Composition

The concentration of NH_4_^+^ and NO_3_^−^ were measured by automated high‐pressure liquid chromatography (HPLC) with Dionex CS16 cation exchange column and AS12 anion exchange column, respectively. The concentration of NO_2_^−^ was measured using colorimetric method (Greenberg et al. 1992) with a Hash, DR/2010 spectrophotometer.

The δ^15^N‐NH_4_^+^ and δ^15^N, δ^18^O‐NO_3_^−^ of nutrient solution were measured using the azide method modified from McIlvin and Altabet 2005 (Semaoune et al. 2012). This two‐step method consists first in transforming the NO_3_^−^ or NH_4_^+^ to nitrite (NO_2_^−^) and secondly in reducing NO_2_^−^ to nitrous oxide (N_2_O) using sodium azide in an acetic acid buffer. For NO_3_^−^, we adapted and used the two‐step cadmium‐azide method (McIlvin and Altabet 2005). The main modification was the use of one granular‐cadmium filled‐column instead of spongy cadmium to convert nitrate to nitrite, to simplify and accelerate the step and to ensure a complete conversion (Shilman and Teplyakov 2007). The volume of each sample was determined to obtain 40 mL of a solution at 5 to 20 µM of NO_3_^−^ in sodium chloride buffer (pH 8.5). From the 40 mL of nitrate passed through the cadmium column, the first 20 mL eluted were discarded and the following 20 mL of nitrite were collected. NH_4_^+^ was oxidized to NO_2_^−^ by hypobromite (BrO^−^) at pH 12 (Zhang et al. 2007). The samples were prepared from 15 nmol of nitrite in vials sealed with Teflon‐lined septa. The nitrites were then reduced to nitrous oxide (N_2_O), by injecting 0.8 mL of azide‐acetic acid buffer with a syringe,. After 15 min, 0.1 mL of NaOH (10 M) was added to stop the reaction. Samplings were made for each point of the kinetics and the analysis of isotopic composition of the nutrient solution was possible for the sample with a concentration above 1.6 μM N L^−1^ (0.1 mg N L^−1^).

The stable isotope composition of N_2_O was determined using the purge‐and‐trap and continuous‐flow isotope ratio mass spectrometry (CF‐IRMS) system (Delta V plus Thermo coupled with Gas Bench II). NO_3_^−^ standards (USGS32, δ^15^N = + 180‰, δ^18^O = + 25.7‰; USGS34, δ^15^N = −1.8‰, δ^18^O = −27.9‰ and USGS35, δ^15^N = + 2.7‰, δ^18^O = + 57.5‰) and NH_4_^+^ standards (IAEA‐N1, δ^15^N = + 0.4‰; USGS25, δ^15^N = −30.4‰; IAEA 305, δ^15^N = 39.8‰) were analyzed to calibrate the method. Internal standard NO_3_^−^ (KNO_3_, δ^15^N = + 4.5‰, δ^18^O = + 25‰) and NH_4_^+^ (NH_4_SO_3_^−^, δ^15^N = 0.3‰), were analyzed to check the linearity of the analysis. The precision of the method was 0.3‰ for δ^15^N and 1.8‰ for δ^18^O for NO_3_^‐^ analysis and 0.5‰ for δ^15^N for NH_4_^+^ analysis.

Plant samples were analyzed for N content and N stable isotope ratios via an elemental analyzer coupled with an isotope ratio mass spectrometer in continuous flow mode (Eurovector‐isoprime). A standard deviation of 0.1‰ was obtained on a randomly selected set (17%) of samples analyzed in triplicate; the precision of apple leaf standard, NIST 1515, was 0.3‰ the δ^15^N mean value being 0.4‰.

### 2.4 Isotopic Fractionation and Discrimination

The N isotope composition of sample is expressed in parts per thousand by

(1) $$\delta_{\text{Nsample}}\left[‰\right]\;=\;\left[\left(R_{\text{sample}}-\;R_{\text{standard}}\right)/R_{\text{standard}}\right)\;*\;10^{3}$$

Where R is the isotopic ratio ^15^N/^14^N or ^18^O/^16^O. The international standards are atmospheric N_2_ and V‐SMOW (Standard Mean Ocean Water) for δ^15^N and δ^18^O measurements, respectively. Isotopic fractionation occurs when isotopes react at different rates causing variation in isotopic composition between product and substrate. The isotopic fractionation during a reaction Substrate (S) → Product (P) is defined as the ratio of the reaction rate for the two isotopes:

$$\alpha =k^{18}O/k^{16O}\text{or}\;k^{15}N/k^{14}N,$$

where *k* is the reaction rate constant for each isotope.

The isotopic enrichment factor *ε*, in ‰ is defined as:

ε=(α−1)×1000.

Using N as an example in a unidirectional, homogeneous, and irreversible reaction under closed system conditions, the isotopic composition of the substrate at time *t* (*δ*_St_) can be calculated by the Rayleigh equation (Mariotti et al. 1981) using the initial isotopic value and the rate of the reaction:

(2) $${\delta N}_{\text{St}}={\delta N}_{S0}+\varepsilon \;*\;\ln\;f$$

Where δN_S0_ is the isotopic composition of the substrate at initial time and *f* = *C*_t_/*C*_0_ with *C*_0_ and *C*_t_ the concentrations of the substrate at time 0 and *t,* respectively. Consequently, isotopic fractionation (*ε*) can be calculated as the slope of the relationship between the substrate *δ*_St_ and the fraction of substrate which has reacted.

The isotopic composition of the product formed during the reaction can be written:

(3) $${\delta N}_{p}={\delta N}_{S0}–\;\varepsilon \;*\;f\;*\;\left(\ln\;f\right)/1-f$$

In this study, the isotope discrimination (Δ) is defined as:

(4) $$\Delta_{S-P}=\;{\delta N}_{\text{source}}–\;{\delta N}_{\text{product}}.$$

It is important to note that in the present experiments, the N source supply was limited. Therefore, Δ cannot be an approximation of *ε*.

## 3 Results

### 3.1 Uptake Kinetics and Growth

**Figure 1:**
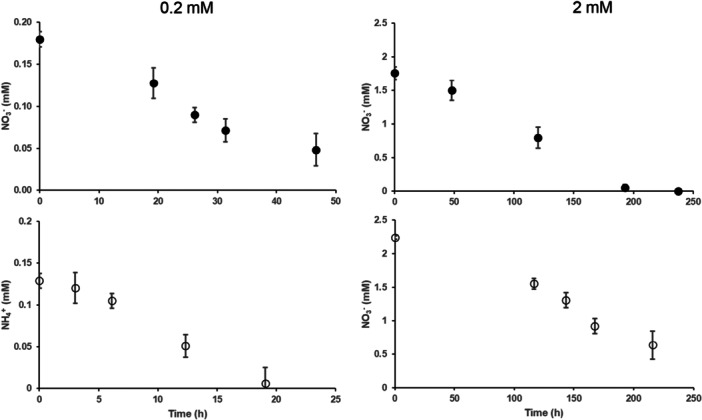
Evolution of NO_3_^−^ or NH_4_^+^ concentrations in the nutrient solution during maize growth with initial concentrations of 0.2 or 2 mM. For each treatment, mean concentration and standard deviation of the 3 replicates are represented.

The N concentration in the nutrient solution, initially set at 0.2 and 2 mM of NO_3_^−^ or NH_4_^+^, decreased over time due to absorption by the plants (Figure 1). The three experimental replicates for each substrate and concentration exhibited similar uptake kinetics. Therefore, they were treated as replicates in subsequent analyses. Maize roots and shoots exhibited greater growth under NO_3_^−^ than NH_4_^+^ for both treatments (Table 1). NH_4_^+^ is known to exert a toxic effect on most plants, with symptoms typically appearing when external NH_4_^+^ concentration exceeds 0.5 mM (Britto and Kronzucker 2002). The significant growth differences observed between the 2 mM NH_4_^+^ and NO_3_^−^ nutrient solutions were likely due to NH_4_^+^ toxicity. During the uptake kinetics, the N content of plants grown with the high substrate concentration (2 mM) increased (from 6.4 ± 0.8 to 21.8 ± 3.5 mg N g plant^−1^ for NH_4_^+^ and from 13 ± 3.1 to 30 ± 2.6 mg N g plant^−1^ for NO_3_^−^). In contrast, this trend was less pronounced at the lower substrate concentration (0.2 mM). The N content of plants grown with the 0.2 mM NO_3_^−^ increased only slightly, from 4.1 ± 1.2 to 5.9 ± 1.2 mg N g plant^−1^, while that of plants grown with 0.2 mM NH_4_^+^ decreased from 5 ± 2.4 to 3.9 ± 1.1 mg N g plant^−1^. This likely reflects the smaller contribution of N uptake relative to the pre‐existing N pool within plant.

**Table 1:** Dry weights (g) of maize plant at the end of uptake kinetics with the yield of N.

	NH_4_^+^				NO_3_^−^			
Plant part	0.2 mM	% N	2 mM	% N	0.2 mM	%N	2 mM	% N
Roots	0.11 ± 0.06	1.5 ± 0.4	0.17 ± 0.04	3.2 ± 0.8	0.22 ± 0.07	1.5 ± 0.1	0.28 ± 0.06	1.8 ± 0.3
Shoots	0.19 ± 0.08	1.2 ± 0.4	0.39 ± 0.2	3.7 ± 1.8	0.26 ± 0.02	1.5 ± 0.2	1.18 ± 0.27	2.3 ± 0.3

*Note:* Means of fresh weight ± SE of the three plants (one plant per container) per treatment, collected in the last point of the uptake kinetics. For NH_4_^+^ uptake kinetics, the plants were 29 day old for 0.2 mM and 37 day old for 2 mM. For NO_3_^−^ uptake kinetics, the plants were 31 day old for 0.2 mM and 38 day old for 2 mM. Table 1 Dry weights (g) of maize plant at the end of uptake kinetics with the yield of N.

### 3.2 Isotopic Fractionation With Net Uptake (*ε*) and Discrimination (Δ)

**Figure 2:**
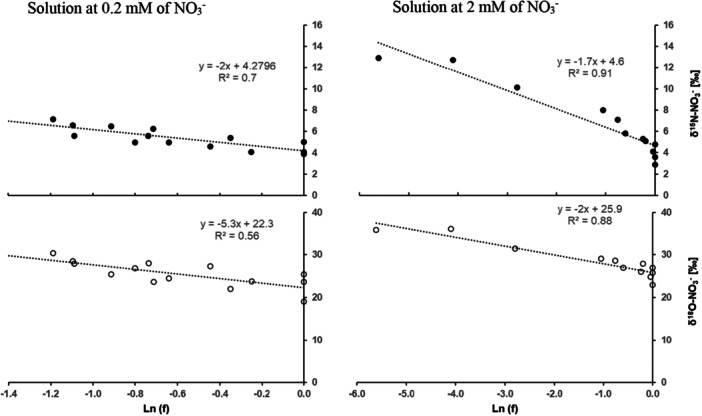
Relationship between δ^18^O and δ^15^N‐NO_3_^−^ of the nutrient solution (‰) and the natural logarithm of the fraction of NO_3_^−^ remaining in solution during the absorption of NO_3_^−^ by maize, in closed system on the left with 0.2 mM and the right 2 mM for nitrate concentration. All points of the three replicates of the experiment were represented for each concentration. ε was defined as the slope of the linear regression.

**Figure 3:**
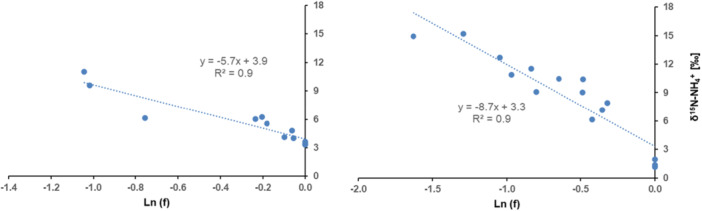
Relationship between δ^15^N‐NH_4_^+^ of the nutrient solution (‰) and the natural logarithm of the fraction of NH_4_^+^ remaining in solution during the absorption of NH_4_^+^ by maize, in closed system. All points of the three replicates of the experiment were represented for each concentration. ε was defined as the slope of the linear regression. [Color figure can be viewed at wileyonlinelibrary.com]

As N was absorbed, the remaining NH_4_^+^ and NO_3_^−^ in nutrient solution became increasingly enriched in ^15^N for the two concentrations (Figures 2 and 3). This study determined the isotopic fractionations for both N (^15^ε) and O (^18^ε) in NO_3_^−^ with the nutrient solution. Linear regressions were used to calculate ^15^ε and ^18^ε values for NO_3_^−^ net uptake (Figure 2). Negative ^15^ε and ^18^ε values were observed, indicating net discrimination against ^15^N and ^18^O. The ^15^ε values for nitrate were not significantly different between the low (0.2 mM; −2‰) and the high (2 mM; −1.7‰) initial concentrations. In contrast, the ^18^ε value was depended on the initial substrate concentration, with −5.3‰ at 0.2 mM and −2‰ at 2 mM. Similarly, the ^15^ε value associated with NH_4_^+^ net uptake was calculated using data from Figure 3. Like NO_3_^−^, NH_4_^+^ uptake also exhibited negative ^15^ε values, indicating net discrimination against ^15^N. A potential explanation for the fractionation observed during NH_4_^+^ uptake, as described in process (3) of the introduction, is the production of ^15^N depleted‐NH_3_ during volatilization, which would lead to ^15^N enrichment of the remaining NH_4_^+^ in the nutrient solution. At a pH above 5, as in this experiment, the fraction of NH_3_ is minimal. Despite its rapid diffusion through the membrane, the impact on the remaining NH_4_^+^ in the solution would be negligible with respect to ^15^ε. Therefore, hypothesis (3) should not be considered in the present study. The ^15^ε values were higher for NH_4_^+^ uptake than for NO_3_^−^ uptake and they were concentration dependent with a lower value at low concentration (−5.7‰) than at high concentration (−8.7‰). Nitrogen isotopic fractionation was observed for both NH_4_^+^ and NO_3_^−^ uptake across the concentration range corresponding to the high and low‐affinity transport systems. Two key trends emerged from the data on N isotopic fractionation: (1) NH_4_^+^ uptake exhibited higher isotopic fractionation than for NO_3_^−^, consistent with previous studies (Yoneyama et al. 2001; Ariz et al. 2011). However, this differs from findings by Evans et al. (1996) and Kolb and Evans (2003) who reported low or negligible isotopic fractionation for NH_4_^+^ uptake, as well as NO_3_^−^ uptake (Yoneyama and Kaneko 1989; Evans et al. 1996). (2) Isotopic fractionation associated with NH_4_^+^ uptake increased with external concentration while a small and concentration‐independent N isotopic fractionation was observed for NO_3_^−^ uptake. These results contrast with studies reporting an increase in ^15^ε associated with NO_3_^−^ uptake as external concentration increase (Mariotti et al. 1982; Kolb and Evans 2003). Consistent with negative ^15^ε values observed for both NH_4_^+^ and NO_3_^−^ nutrition, plants were depleted in ^15^N relative to their N source, resulting in positive discriminations (Δ_S‐P_) values (Table 2). In NO_3_^−^ media, Δ_S‐P_ value varied between 4 ± 1.5‰ and 3 ± 0.9‰ at low concentration and 1.5 and −0.1‰ at high concentration. In NH_4_^+^ media, Δ_S‐P_ value varied between 3.6 ± 0.5‰ and 2.8 ± 0.2‰ at low concentration and from 5.3 ± 0.4‰ to 2.8 ± 0.7‰ at high concentration. For both NH_4_^+^ and NO_3_^−^ nutrition, and the two treatments, Δ_S‐P_ values decreased with decreasing concentration in the solution and increase of the net uptake.

**Table 2:** Evolution of discrimination between source and plant δ^15^N (Δ_S‐P_) along the uptake kinetics (*t*_o_ to *t*_4_).

		Δ_S‐P_ (‰)				
		*t*_o_	*t*_1_	*t*_2_	*t*_3_	*t*_4_
NO_3_^−^	0,2 mM	4 ± 1.5	4.5 ± 1	3.6 ± 0.5	3.1 ± 0.5	3 ± 0.9
	2 mM	1.5 ± 0.4	1.8 ± 0.6	1.6 ± 1.1	0.9 ± 0.2	−0.1 ± 0.2
NH_4_^+^	0,2 mM	3.6 ± 0.5	2.6 ± 0.6	3.3 ± 0.6	3.3 ± 0.5	2.8 ± 0.2
	2 mM	5.3 ± 0.4	6.7 ± 0.7	6.5 ± 0.4	5.9 ± 1.1	2.8 ± 0.7

*Note:* Container 1, 2 and 3 were the replicates of the experiment for each treatment.

### 3.3 Isotopic Composition of NO_3_^−^ Roots

**Figure 4:**
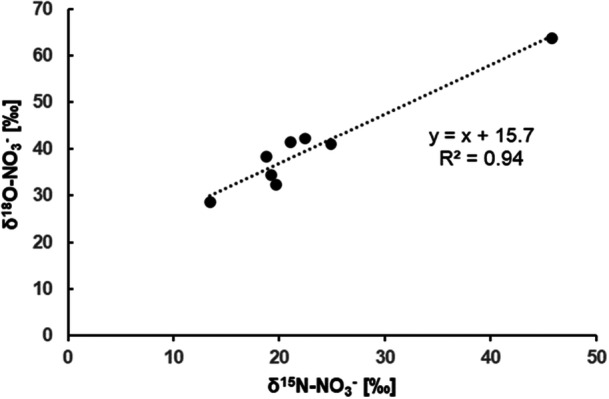
Relationship between the N and O isotopic composition of NO_3_^−^ in roots of Maize grown under 0.2 and 2 mM concentration of NO_3_^−^. The two outliers were excluded of the linear regression.

The NO_3_^−^ pool in roots was significantly enriched in ^15^N and ^18^O compared to the NO_3_^−^ in nutrient solution (Table 3). This enrichment was more pronounced at low concentration than at high concentration at the beginning of the experiment (*t*_0_). By the end of the experiment (*t*_4_), δ^15^N values of NO_3_^−^ at the high substrate concentration had increased and close to the δ^15^N values at low concentration except for container 1. Subsequently, the outlier (container 1, *t*_4_, 0.2 mM) was therefore excluded for the calculation of mean. For N, at low concentration, the mean value of δ^15^N‐NO_3_^−^ within roots was 21.5 ± 3.9‰ at *t*_0_ and 19.5 ± 0.3‰ at *t*_4_. At high concentration, the mean value of δ^15^N‐NO_3_^−^ within roots was 12.4 ± 2.7‰ at *t*_0_ and 19.4 ± 1.5‰ at *t*_4_. For oxygen, at low concentration, the mean value of δ^18^O‐NO_3_^−^ within roots was 45.5 ± 6.7‰ at *t*_0_ and 43.5 ± 17.6‰ at *t*_4_. At high concentration, δ^18^O‐NO_3_^−^ within roots was 32.6 ± 5.6‰ at *t*_0_ and 36.1 ± 6.7‰ at *t*_4_. A strong correlation between δ^15^N and δ^18^O in the NO_3_^−^ pool within roots was observed, with linear regression (slope of 1 and *r*² = 0.94) (Figure 4).

**Table 3:** Nitrate isotopic composition for roots extract and nutrient solution at the beginning (*t*_0_) and at the end (*t*_4_) of the NO_3_^−^ experiments.

	0.2 mM				2 mM			
	NO_3_^−^ roots		NO_3_^−^ source		NO_3_^−^ roots		NO_3_^−^ source	
	δ^15^N [‰]	δ^18^O [‰]	δ^15^N [‰]	δ^18^O [‰]	δ^15^N [‰]	δ^18^O [‰]	δ^15^N [‰]	δ^18^O [‰]
T0	21.5 ± 3.9	45.5 ± 6.7	3.8 ± 1.0	25.2 ± 2.1	12.4 ± 2.7	32.6 ± 5.6	4.4 ± 0.6	22.7 ± 3.3

### 3.4 Modeling

#### 3.4.1 Simulating ε Resulting from Efflux

The process of net N uptake unequivocally results in significant isotopic fractionation. According to the model proposed by Mariotti et al. (1982), isotopic fractionation with net uptake is influenced by the efflux of inorganic N enriched in ^15^N; which arises from assimilation processes in the roots. In this model, variations in δ^15^N and δ^18^O in the nutrient solution are only attributed to the efflux of ^15^N enriched NO_3_^−^ from roots with no isotopic fractionation occurring during uptake. The N and O isotopic fractionation would depend on the efflux rates and the isotopic composition of the effluxed NO_3_^−^. To test this hypothesis, we calculated efflux rates required to account for the observed ^15^ε values in nutrient solution. These calculated efflux rates were subsequently used to predict trends in δ^18^O‐NO_3_^−^ within the nutrient solution. The predicted ^18^ε values were then compared with experimentally measured values.

**Figure 5:**
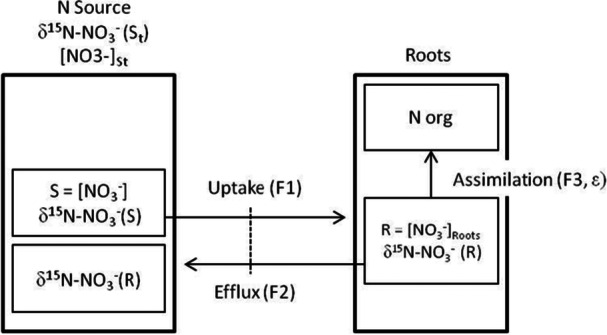
Schematic representation of N circulation between roots and N source in closed system. The isotopic composition in the source at any time (δ^15^N‐NO_3_^−^ (*S_t_*)) and nitrate concentration (*S_t_*) depend on initial concentration (*S*_0_) and isotopic composition (δ^15^N‐NO_3_^−^ (*S*_0_)) in this reservoir and the portion of nitrate efflux. Amount of N and the isotopic composition of the efflux depend on of nitrate assimilated and the isotopic fractionation generated (*ε*).

The nitrate pools, fluxes, and isotopic compositions in closed system are summarized in Figure 5. The flux rates *F*_1_, *F*_2_ and *F*_3_ represented the uptake, the flux and the assimilation. During uptake by plant, the substrate concentration of NO_3_^−^ (*S*) gradually decreases. *S* = *S*_0_–*F*_1_*Δ*t* with Δ*t* the delay between two successive samplings. *S*_t_ represents the concentration of NO_3_^−^ source at each sampling point resulting from *F*_1_ and *F*_2_, *S*_t_ = *S*_0_–*F*_1_* Δ*t* + *F*_2_* Δ*t* and so

(5) $${\text{S}}_{t}\;=\;S+F_{2}*\text{D}\text{t}.$$

It was hypothesized that only the process of assimilation fractionates and the processes of uptake and efflux were considered as no fractionating transport of NO_3_^−^ between roots and nutrient solution. δ^15^N‐NO_3_^−^(*S*) is in consequence not modified and corresponds to δ^15^N‐NO_3_^−^(*S*_*t*−1_) for each point of uptake kinetics. δ^15^N‐NO_3_^−^(*S_t_*) is determined by the isotopic composition of NO_3_^−^ source remaining in solution after uptake and the isotopic composition of NO_3_^−^ efflux from roots (*R*) as follows:

(6) $$\delta^{15}N-{{\text{NO}}_{3}}^{-}\left(S_{t}\right)=\left(\delta^{15}N-{{\text{NO}}_{3}}^{-}\left(S_{t-1}\right)*S+\delta^{15}N-{{\text{NO}}_{3}}^{-}\left(R\right)*F_{2}\right)/(S+F_{2}*\text{Dt})$$

The rates of efflux necessary to induce the variation of δ^15^N‐NO_3_^−^ observed in nutrient solution can be calculated from Equations (4) and (5) by:

(7) $$F_{2}=(\text{St}\;*\;\left(\delta^{15}N-{{\text{NO}}_{3}}^{-}\left(S_{t}\right)\;-\;\delta^{15}N-{{\text{NO}}_{3}}^{-}\left(S_{t-1}\right)\right)/\left(\delta^{15}N-{{\text{NO}}_{3}}^{-}\left(R\right)\;-\;\delta^{15}N-{{\text{NO}}_{3}}^{-}\left(S_{t-1}\right)\right)\;*\text{Dt})$$

The effluxes were simulated between each point of the uptake kinetics for the three replicate containers of the two concentrations. We used the experimentally measured values of δ^15^N‐NO_3_^−^(*S_t_*), δ^15^N‐NO_3_^−^(*S*_*t*−1_) and δ^15^N‐NO_3_^−^(*R*) and of the nitrate concentrations *S_t_*.

**Figure 6:**
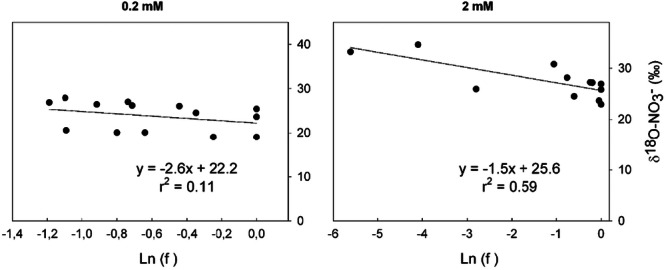
Simulation of δ^18^O‐NO_3_^−^ along the uptake kinetics according to the efflux calculated from δ^15^N‐NO_3_^−^ variations in nutrient solution. All points of the three replicates of the experiment were represented for each concentration. *ε* was defined as the slope of the linear regression.

At high substrate concentration, the calculated efflux rates *F*_2_ decreased progressively along the kinetics ranging between 61 and 0.6 µg N h^−1^. At low substrate concentration, the efflux rates *F*_2_ were consistently low and ranged between 11.8 and 0.0 µg N h^−1^ with no discernible trend. The greater efflux observed at high substrate is consistent with nitrate reductase (NR) exposure to a larger N pool (*F*_3_ < *F*_1_). Previous studies calculated inorganic N efflux rates relative to plant biomass (Teyker et al. 1988; Ter Steege et al. 1999). When expressed relative to plant biomass, the present data align with these reported efflux rates. Using the calculated *F*_2_ values, δ^18^O‐NO_3_^−^ simulations were generated (Figure 6). Similar to the experimental data, the simulated ^18^ε value for δ^18^O‐NO_3_^−^ depended on the initial substrate concentration. At low substrate concentration, the simulated ^18^ε value was −2.6‰, compared to −1.5‰ at high substrate concentration. A low correlation coefficient (*r* ^2^ = 0.11) was determined for the 0.2 mM treatment, primarily due to the influence of container 1. While the values measured for container 1 were internally consistent throughout the kinetics, they were distinctly lower than those for the other replicates. Excluding these values did not alter the ^18^ε value but substantially improved the correlation coefficient (*r* ^2^ = 0.72). Nevertheless, the simulated ^18^ε values were lower than those calculated from the experimental data (−5.3 and −2.1‰ at low and high concentration, respectively).

#### 3.4.2 Whole plant δ^15^N

In a closed system, the extent of discrimination associated with isotopic fractionation during net uptake (Δ_S‐P_, Equation 4) depends on the fraction of N remaining in the solution. It is generally assumed that when plants completely absorb the N source, the plant δ^15^N should closely match the δ^15^N of the source. However, this relationship becomes more complex when the internal N pool within the plant is large relative to the N supply, as the new isotopic composition of N source may have negligible impact on the plant's δ^15^N. Additionally, other processes, such as the efflux of organic N, could potentially influence the plant δ^15^N. To evaluate whether ^15^ε value is the primary driver of discrimination between plant and soil δ^15^N, we excluded the influence of other processes. In this analysis, total N in plants was considered a product of the net N uptake process, and the experimentally determined ^15^ε value was used as the isotopic fractionation coefficient associated with net uptake. Under the experimental conditions, because N concentration was not zero at initial time of the experiment, plant δ^15^N was expressed as composite value incorporating the isotopic composition of the pre‐existing N stocks and the newly absorbed by plants. As N is absorbed by plant, the δ^15^N values of the resulting pool within plant (product) can be described using a Rayleigh equation. Based on Equations (2 and 3), the progression of plant δ^15^N can be written as:

(8) $$\delta^{15}N_{\text{plant}}\left(t\right)\;=\;\left[\left(\delta^{15}N_{\text{plant}}\left(t_{0}\right)\;*\;P\left(t_{0}\right)+\left(\delta^{15}N_{S0}–\;\varepsilon_{\text{net}\;\text{uptake}}*\;f\;*\;\ln\;f/\left(1-f\right)\right)\;*\;S_{0}\left(1-f\right)\right)\right]/\left(P\left(t_{0}\right)+S_{0}\left(1-f\right)\right),$$

where P(*t*_0_) was the total concentration of N in plant at initial time and δ^15^N_plant_(*t*_0_) its isotopic composition. *f* = *C*_t_/*C*_0_ with *C*_t_ and *C*_0_ the concentrations of the substrate at time *t* and 0, respectively.

**Figure 7:**
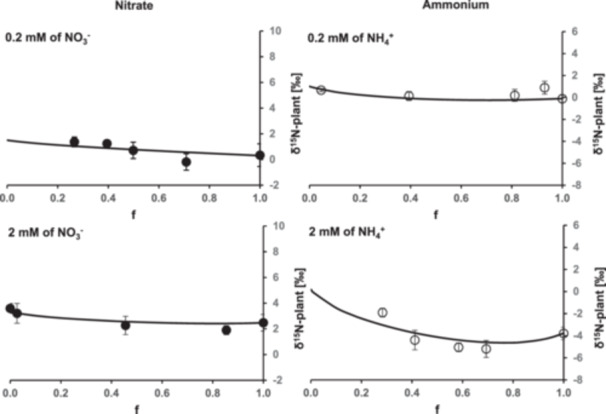
Simulation of plant δ^15^N during N uptake kinetics with ε associated with uptake and a mixture of N pre‐existing stocks and of N absorbed by plant (solid curve). Comparison with experimental data (filled circles). The isotopic composition of N source at initial time is represented by dashed line. Each point represents the mean of three measurements ± SD.

Results of plant δ^15^N obtained from both simulation (Equation 8) and laboratory experiments are presented in Figure 7. For both NH_4_^+^ and NO_3_^−^ forms at 0.2 and 2 mM, the simulated plant δ^15^N closely matched the experimentally observed evolution of plant δ^15^N during uptake kinetics. Following the complete consumption of N by the plant, the simulations indicated that the Δ_S‐P_ was greater at low concentrations compared to high concentrations at the end of the kinetics.

## 4 Discussion

### 4.1 Mechanisms of Nitrate Isotope Fractionation

Two mechanisms have been proposed to explain isotopic fractionation during NO_3_^−^ uptake. The first hypothesis involves the intervention of different concentration‐dependent carrier systems that may exhibit varying discrimination against ^15^N. However, an analysis of the present data in conjunction with previous studies supports two key conclusions similar to those drawn by Evans (2001): First, no N isotopic fractionation was observed for a wide range of concentrations, suggesting that intrinsic isotopic fractionation is not associated with either high‐ or low‐affinity transport systems. Second, the measured ^15^ε values were not directly correlated with external N concentrations, indicating N concentration alone does not determine isotopic fractionation. Thus, this first hypothesis can be excluded.

The second hypothesis attributes isotopic discrimination to the efflux of unassimilated NO_3_^−^ enriched in ^15^N, which occurs when the rate of uptake exceeds that of assimilation (Mariotti et al. 1982; Robinson et al. 1998; Comstock 2001). Subsequent studies have reported that NO_3_^−^ reduction also leads to ^18^O enrichment with ε^18^O: ε^15^N ratios from 0.5 to 2.3 during denitrification (Böttcher et al. 1990; Bourbonnais et al. 2015), and approximately 1 during phytoplankton assimilation (Granger et al. 2004). In the current study, a ε^18^O: ε^15^N ratio of 0.8 for the NO_3_^−^ pool in roots, indicating slightly higher ^18^ε. An isotope mixing equation was employed to test whether efflux of the unassimilated NO_3_^−^ from roots could account for the observed ^15^N and ^18^O enrichment of the substrate in the solution. Indeed, a low efflux of NO_3_^−^ was found to induce ^15^N enrichment in the nutrient solution, driven by the significant ^15^N enrichment during assimilation. This efflux could also generate higher O isotopic fractionation at low substrate concentrations than at high concentrations, consistent with the experimental observations. However, the simulated ^18^ε values were lower than those calculated from the experimental data. This discrepancy could be explained by evaporation during the experiment, which enriched water in ^18^O, as well as oxygen exchange between water and NO_2_^−^ during the azide method, as reported by McIlvin and Altabet 2005. These findings suggest that efflux may be the primary driver of ^15^N and ^18^O enrichment in external NO_3_^−^ across a broad range of concentration. Logically, an increase in efflux would result in higher enrichment of the N source. However, in the high concentration experiment, where calculated efflux rates were higher, the ^15^ε remained similar to that observed at low concentration. This could be attributed to differences in δ^15^N‐NO_3_^−^ within roots between the two concentrations. At the high concentration, the initial root δ^15^N value of NO_3_^−^ was slightly lower than at low concentration. As a result, for an equivalent efflux rate, the ^15^N enrichment would be relatively higher in low concentration. The higher efflux at high concentration likely offset the difference in root δ^15^N‐NO_3_^−^ between the two concentrations, resulting similar ^15^ε values. Thus, the extent of ε appears to be driven by the rate of efflux and the isotopic composition of NO_3_^−^ in roots. Understanding the occurrence and extent of isotopic fractionation associated with net uptake is challenging, as efflux rates exhibit complex regulatory patterns, and the isotopic composition of NO_3_^−^ in roots is influenced by multiple processes. Here, we aim to identify the primary factors influencing the two drivers.

### 4.2 Efflux Regulation by Root Nitrate Content

Previous studies hypothesized that isotopic discrimination during NO_3_^−^ uptake occurs exclusively at high concentrations, where nitrate uptake rates exceed assimilation and efflux was possible (Mariotti et al. 1982; Kolb and Evans 2003). However, N isotopic fractionation was observed across a wide range of external concentrations, indicating that substrate concentration alone does not primarily regulate the efflux. Physiological studies on higher plants demonstrated NO_3_^−^ efflux occurs over a broad range of external concentration and is largely independent of these concentrations (Ter Steege et al. 1999). Furthermore, efflux rates declined when root NO_3_^−^ content decreased (Teyker et al. 1988; Ter Steege et al. 1999), suggesting that NO_3_^−^ efflux is regulated by root cytoplasmic nitrate concentration (Ter Steege et al. 1999). The present results support this hypothesis, as the highest calculated efflux rates were observed in plants grown under high NO_3_^−^ concentration, which correlated with higher root NO_3_^−^ contents. In addition, previous studies reported that isotopic discrimination decreased with plant age (Mariotti et al. 1982; Kolb and Evans 2003). It has been attributed to increased NR activity in older plants, as rapid assimilation could limit efflux (Mariotti et al. 1982). However, more recent findings found no evidence of increased NR activity with plant age but lower root NO_3_^−^ contents were reported in older plants when compared to younger ones (Kolb and Evans 2003).

### 4.3 Influence of External Nitrate Concentration on Tissue Isotopic Composition

Root NO_3_^−^ content thus emerges as a critical regulator of efflux rates. Plant species, external substrate concentration and growth conditions may influence the internal NO_3_^−^ pool content and efflux rates. These factors may account for the wide range of ^15^ε values reported in the literature (Mariotti et al. 1982; Yoneyama and Kaneko 1989; Evans et al. 1996; Yoneyama et al. 2001; Kolb and Evans 2003; Ariz et al. 2011; Kalcsits et al. 2015). The isotopic composition of NO_3_^−^ in plant was influenced by external NO_3_^−^ concentrations. Plants grown at low concentrations exhibited NO_3_^−^ pool more enriched in ^15^N and ^18^O than plants grown at high concentration. Moreover, at high concentration, the N and O isotopic composition of NO_3_^−^ increased over the course of the kinetics. Taken together, these observations indicate that lower external substrate concentration results in higher N and O isotopic composition in root NO_3_^−^. This relationship may be explained by the combined effects of NO_3_^−^ reduction by NR and its storage in vacuole, which does not undergo assimilation, on root isotopic composition. NO_3_^−^ storage decreased as external substrate concentrations decreased, and isotopic enrichment driven by NR activity became more pronounced when NO_3_^−^ pool were smaller. At high concentration, stored NO_3_^−^ may represent a larger fraction of the NO_3_^−^ pool, partially offsetting the isotopic effect associated with NR activity.

### 4.4 Ammonium Uptake and Isotopic Fractionation

NH_4_^+^ uptake and assimilation are expected to follow similar patterns to those described by the model of efflux. Similar to NR, glutamine synthetase induces a substantial isotopic fractionation (ε = −15‰) resulting in ^15^N‐enrichment in unassimilated NH_4_^+^ within roots. NH_4_^+^ is known to exert toxic effects on most plants, with symptoms typically manifesting when external NH_4_^+^ concentration exceeds 0.5 mM (Britto and Kronzucker 2002). Recent studies demonstrate that substantial NH_4_^+^ efflux occurs at elevated concentration (20 mM), coinciding with inhibited root elongation (Li et al. 2010). As external NH_4_^+^ concentration increases, efflux of ^15^N‐enriched NH_4_^+^ from roots is expected to rise, leading to higher ^15^ε values. This is consistent with the present findings, which demonstrated that ^15^ε associated with NH_4_^+^ uptake increased with substrate concentration, while root growth was reduced at higher concentration. Unlike NO_3_^−^, NH_4_^+^ is not stored and rapidly assimilated regardless of external concentration, resulting in relatively constant internal concentration; therefore, assimilation likely plays a minor role in ^15^ε variations. NH_4_^+^ efflux appears to be associated with its toxic effects; consequently, external concentration and plant sensitivity to NH_4_^+^ are likely the main factors influencing the isotopic fractionation.

### 4.5 Plant‐Source Discrimination and the Role of Efflux

For both NO_3_^−^ and NH_4_^+^, plant absorption induces an isotopic fractionation, resulting in discrimination between the δ^15^N of the source and that of the plant. Negative ^15^ε values indicate that the substrate becomes enriched in ^15^N relative to the product. During net N uptake, negative ^15^ε values suggest ^15^N depletion in the plant and ^15^N enrichment in the nutrient solution. Consistently, positive discriminations (source δ^15^N‐ plant δ^15^N) were observed for both inorganic N forms (NO_3_^−^ and NH_4_^+^) at 0.2 and 2 mM concentrations. Some studies suggested that the efflux of organic N may influence plant δ^15^N, contributing to differences between δ^15^N in N source and that of the plant (Kolb and Evans 2003; Comstock 2001; Robinson et al. 1998). Kolb and Evans (2003) reported that no amino acids were detected in the nutrient solution but hypothesized that more complex organic N might be released via efflux. We used an isotopic mixing model to evaluate the influence of ^15^ε associated with inorganic N net uptake on plant δ^15^N. Plant δ^15^N was treated as a product of net uptake, characterized by isotopic fractionation values observed experimentally. This supports the conclusion that N efflux had a negligible impact. For NO_3_^−^ and NH_4_^+^ at both 0.2 and 2 mM, the simulation of plant δ^15^N was consistent with the experimental measurements. This further confirms that organic N efflux had a negligible on plant δ^15^N. The isotopic fractionation during uptake is the main factor driving the isotopic discrimination. However, other parameters must be considered to fully explain the observed patterns of discrimination.

### 4.6 Influence of Plant Nitrogen Content on Discrimination

It is generally admitted that if an organism exhausts an N source, the δ^15^N of the organism will be very close to that of the N source. However, in the present experiment, this relationship is more complex because the initial N content in plants was not zero. Consequently, plant δ^15^N was expressed as composite values that integrates the isotopic composition of pre‐existing N stocks and N absorbed by plants during the experiment. The simulation revealed that the isotopic discrimination between N source and plant (Δ_S‐P_) at any given time depended on the amount of N supplied relative to plant N content and the initial difference between δ^15^N of the plant and the solution. Consistent with our observations, when the quantity of supplied N was small compared to the plant N content (e.g., 0.2 mM), Δ_S‐P_ remained elevated even after complete absorption of the supplied N. Similarly, when the initial difference between plant δ^15^N and source δ^15^N was large (e.g., 2 mM), a higher quantity of source N was required for the plant δ^15^N to converge with that of the source. Nonetheless, changes in the δ^15^N induced corresponding changes in plant δ^15^N. Consequently, while plant δ^15^N can act as an integrator of source δ^15^N over time, it is unsuitable as a precise tracer of the source δ^15^N.

### 4.7 Transferability to Field Conditions: The Role of Mycorrhizae

While our hydroponic experiments were designed to isolate intrinsic plant mechanisms of nitrogen isotope fractionation, most terrestrial plants—including maize—acquire nitrogen in association with mycorrhizal fungi. These symbionts often dominate nutrient uptake by accessing organic and inorganic N pools in the soil and transferring transformed N compounds to the host plant (Smith and Read 2008; Hobbie and Högberg 2012; Craine et al. 2015; Gorka et al. 2019). Such mediation can alter both nitrogen source preference (NO₃^−^ vs. NH₄⁺) and the magnitude of isotope fractionation, since the fungal partner can discriminate during uptake, assimilation, and translocation to the root.

The relative contributions of fungal and root pathways vary with environmental context, plant genotype, and soil N form (Hogan et al. 2023). In some cases, mycorrhizal fungi transfer already‐assimilated, ¹⁵N‐depleted compounds to the plant; in others, they preferentially retain ¹⁵N‐enriched compounds within their hyphae. Consequently, foliar δ¹⁵N in field‐grown plants reflects a composite of fungal and plant processes, rather than plant uptake alone.

Therefore, the fractionation factors quantified in this study should be regarded as mechanistic baselines, describing intrinsic plant discrimination in the absence of symbiosis. These values provide the necessary foundation for integrating mycorrhizal and microbial effects into isotopic models of plant N acquisition under natural conditions.

## 5 Conclusion

This study provides new insights into the mechanisms governing nitrogen isotope fractionation during maize uptake of ammonium (NH₄⁺) and nitrate (NO₃^−^) under controlled hydroponic concentrations. Isotopic fractionation occurred during both N forms, but with distinct patterns. NO₃^−^ uptake exhibited low, concentration‐independent ¹⁵N fractionation, whereas NH₄⁺ uptake exhibited stronger, concentration‐dependent isotopic discrimination. These contrasting behaviors suggest that isotopic discrimination is not solely determined by transporter specificity but also shaped by the balance between assimilation and efflux processes.

A key finding of this study is the role of NO₃⁻ efflux in contributing to ¹⁵N and ¹⁸O enrichment in the external solution. This highlights that both uptake and efflux must be considered when interpreting plant δ¹⁵N. The magnitude of discrimination between plant and source δ¹⁵N depends on substrate concentration, the isotopic composition of the source, and the relative rates of uptake and assimilation.

For NH₄⁺, higher fractionation at elevated concentrations suggests that toxicity‐induced efflux may contribute to ¹⁵N enrichment in the residual pool, consistent with known physiological stress responses.

These results establish a mechanistic baseline for understanding isotopic fractionation during N uptake under controlled conditions. However, their transferability to field environments requires caution, as N acquisition in natural systems is largely mediated by mycorrhizal and microbial partners that modify both N form and isotopic composition before transfer to the plant. In this context, the fractionation factors identified here should be viewed as reference values describing intrinsic plant processes, which can be further modulated in soil ecosystems.

Overall, this study advances our understanding of nitrogen isotope behavior during plant uptake and provides a foundation for refining δ^15^N‐based interpretations in ecology and agriculture. Future studies should investigate how plant functional traits, soil N dynamics, and symbiotic associations regulate isotopic discrimination to better link laboratory‐based mechanisms with field‐scale isotope patterns.

## Data Availability

The data that support the findings of this study are available on request from the corresponding author. The data are not publicly available due to privacy or ethical restrictions.
